# Citrus Fruit Intake Substantially Reduces the Risk of Esophageal Cancer: A Meta-Analysis of Epidemiologic Studies

**DOI:** 10.1097/MD.0000000000001390

**Published:** 2015-10-02

**Authors:** Anqiang Wang, Chengpei Zhu, Lilan Fu, Xueshuai Wan, Xiaobo Yang, Haohai Zhang, Ruoyu Miao, Lian He, Xinting Sang, Haitao Zhao

**Affiliations:** From the Department of Liver Surgery, Peking Union Medical College Hospital, Chinese Academy of Medical Sciences and Peking Union Medical College, Beijing, China (AW, CZ, LF, XW, XY, HZ, LH, XS, HZ); and Liver Center and The Transplant Institute, Department of Medicine, Beth Israel Deaconess Medical Center, Harvard Medical School, Boston, MA (RM).

## Abstract

Many epidemiologic studies indicate a potential association between fruit and vegetable intake and various cancers. The purpose of this meta-analysis is to investigate the association between citrus fruit intake and esophageal cancer risk. The authors conducted a comprehensive search on PubMed, EMBASE, and the Cochrane Library from inception until July 2014. Studies presenting information about citrus intake and esophageal cancer were analyzed. The authors extracted the categories of citrus intake, study-specific odds ratio or relative risk, and the *P* value and associated 95% confidence intervals for the highest versus lowest dietary intake of citrus fruit level. The association was quantified using meta-analysis of standard errors with a random-effects model. Thirteen case–control studies and 6 cohort studies were eligible for inclusion. Citrus intake may significantly reduce risk of esophageal cancer (summary odds ratio = 0.63; 95% confidence interval = 0.52–0.75; *P* = 0), without notable publication bias (intercept = −0.79, *P* = 0.288) and with significant heterogeneity across studies (*I*^2^ = 52%). The results from epidemiologic studies suggest an inverse association between citrus fruit intake and esophageal cancer risk. The significant effect is consistent between case–control and cohort studies. Larger prospective studies with rigorous methodology should be considered to validate the association between citrus fruits and esophageal cancer.

## INTRODUCTION

Esophageal cancer, including squamous cell carcinoma (SCC) and esophageal adenocarcinoma (EAC), is a serious malignancy with a poor prognosis in the majority of cases.^1,2^ SCC is the predominant form of esophageal carcinoma worldwide, but a shift in epidemiology has been seen in some countries and regions like Australia, UK, USA, and western Europe, where the incidence of EAC has exceeded that of SCC.^3^ Every year, >450,000 people worldwide are diagnosed with esophageal cancer and the incidence is rapidly increasing.^3,4^ It is the eighth most common cancer, and the sixth most common cause of cancer-related deaths worldwide with developing nations making up >80% of total cases and deaths.^5,6^ The mortality from these cancers is high and the response to treatments during advanced stages is poor, so effectively reducing the chances of exposure to relative risk factors will have an important impact on the incidence of esophageal cancer.

Cigarettes, red meat, alcohol, hot tea, pickled vegetables, low intake of fresh fruits and vegetables, and low socioeconomic status are associated with a higher risk of SCC.^7–10^ Barrett esophagus is clearly recognized as a risk factor for EAC, with other factors including gastroesophageal reflux disease, acid-suppressive medication use, obesity, tobacco use, and processed meat.^11–14^ Some foods can reduce the incidence of esophageal cancer.^9,15–18^ Many researchers conducted meta-analyses on diet and esophageal cancer. The study by Coleman et al^19^ suggested that dietary fiber may protect against esophageal carcinogenesis, especially esophageal adenocarcinoma. Zhu et al^20^ found that meat consumption is associated with the risk of esophageal cancer. The intake of red meat is likely to increase the esophageal SCC risk and the processed meat may increase esophageal adenocarcinoma risk; however, the consumption of fish may not be associated with esophageal cancer incidence. This phenomenon may be explained by the effects of various micronutrients such as folate, B vitamins, antioxidants, lutein, and carotenoids.^21–24^

Citrus fruits include oranges, tangerines, grapefruits, lemons, and limes. They include several components, including flavonoids, folate, carotenoids, and vitamin C,^25-26^ which have protective effects against cancer. Previous studies have suggested that citrus intake may improve the incidence of various cancers including pancreatic, breast, and prostate cancers.^27–29^ Consequently, we hypothesize that citrus intake is associated with a reduced risk of esophageal cancer. Epidemiologic evidence from cohort and case–control studies on this association has not yet been summarized. Therefore, we conducted a meta-analysis to explore this hypothesis.

## STUDY CHARACTERISTICS

### Search Strategy

A computerized search of the English language literature on citrus fruits and esophageal cancer yielded no relevant publications from inception to July 2014. We, therefore, decided to use the key words “fruit” and “citrus.” The search terms were ([esophagus] OR [esophageal]) AND ([cancer] OR [tumor] OR [carcinoma]) AND (‘citrus’itrus OR ‘fruit’ruits). We limited the search to human adults without language restrictions. We searched the 3 major electronic databases: PubMed, EMBASE, and The Cochrane Library. Additionally, we reviewed the references from retrieved articles for additional studies. Furthermore, ethical approval was not necessary because our article is a review.

### Study Selection

The included studies^29^ had to be epidemiologic studies such as case–control and cohort studies. The studies concerning human that addressed the association between citrus intake and incidence of esophageal cancer were collected; however, if the study provides no original data or insufficient information on the odds ratio (OR) or relative risk (RR), and their corresponding 95% confidence intervals (CIs), we excluded it. The studies not measuring the intake of citrus fruits or citrus juice at the individual level are not eligible. The instrument of assessment of citrus intake is questionnaire. Two independent reviewers read the abstracts or full-text articles to assess the eligibility of studies in a standardized manner. We resolved the disagreement by consensus.

### Data Abstraction

We extracted important information from all eligible studies. They included study design, country of origin, years of publication, origin of control, number of cases and control, sex distribution, types of citrus fruits, types of cancer, comparison of exposure level, and potential confounding variables adjusted. The estimates of OR/RR, their associated 95% CIs, and *P* values were also extracted by us. If separate researches based on the same population were published, we selected the article containing more complete information for inclusion.

### Statistical Analyses

We extracted the study specific OR/RR and 95% CIs for highest versus lowest intake of citrus fruits from every study. And we calculated the standard error (SE) of the log OR/RR by using the following equation: SE = (ln[OR/RR_upper − ln OR/RR_lower]) ÷ 3.92. Then, we summarized the overall OR and CI by using general variance-based methods^30^ of RevMan 5.0. For studies that provided OR/RR by cancer subtypes,^15,31^ we used a random-effects model to obtain a pooled estimate from the individual study (Table 1). We adopted the Newcastle-Ottawa Scale to evaluate research quality and defined them as high, middle, and low quality by score 7 to 9, 4 to 6, 1 to 3, respectively. The Grades of Recommendation, Assessment, Development, and Evaluation working group system of rating quality of evidence also were used to evaluate the research quality.

**TABLE 1:**
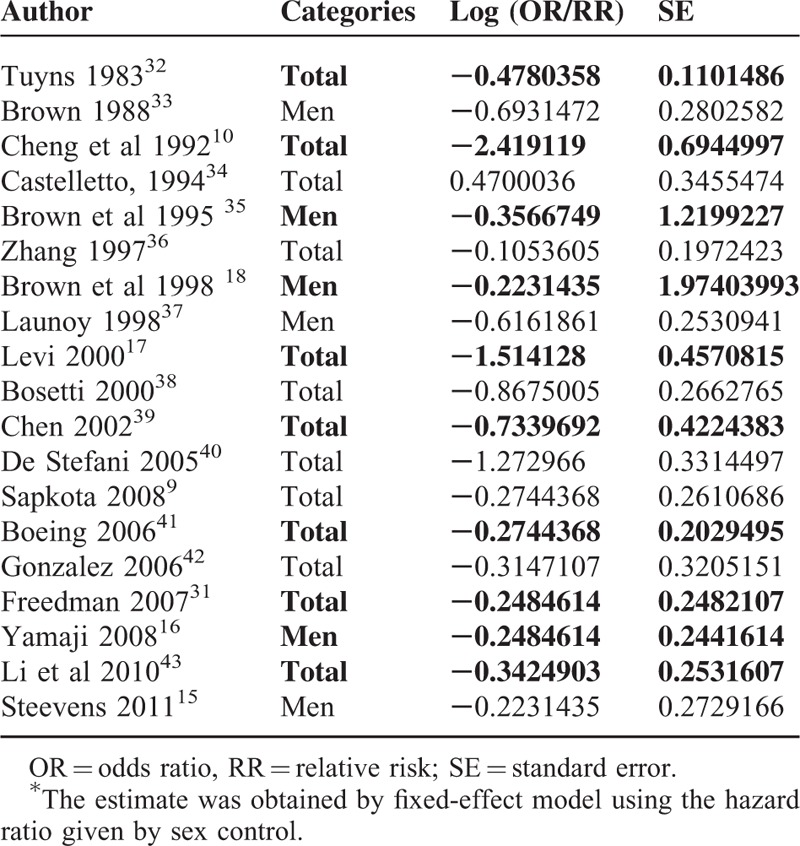
Logarithmic OR or RR (Log[OR/RR]) and Its SE for the Meta-Analysis

The value of *I*^2^ was used to evaluate the extent of heterogeneity derived from study differences rather than chance.^44^ The smaller value *I*^2^ suggested less obvious heterogeneity. We used the random-effects model to calculate the summary OR and its 95% CI^45^ with suspecting heterogeneity. We evaluated the impact of the changes on pooled ORs by study design, cancer subtypes, geographical location, source of controls, research quality, and some adjusted confounders such as alcohol and body mass index as prior hypotheses to explain heterogeneity through subgroup analyses and meta-regression analyses. Sensitivity analyses were conducted by removing 1 study from all studies to evaluate the impact on the pooled ORs and heterogeneity. We can, therefore, evaluate whether the results are stable. In an attempt to detect publication bias, we visually examined asymmetry in a funnel plot. We conducted Begg and Egger test to assess whether there is an obvious publication. We considered the funnel plot to be asymmetrical if the intercept of the regression line deviated from zero with *P* < 0.10. If the test suggests an obvious publication bias, we would conduct the trim and fill analysis to further verify.

We used the Cochrane Collaboration software (Oxford, UK) to analyze the extracted data with fixed or random-effects model analysis.^46^ STATA (StataCorp, College Station, TX) was used to conduct the Egger and Begg regression asymmetry test by using the metabias command.^47^ We conducted the trim and fill analysis to observe whether the results are stable and evaluate the publication bias.

## RESULTS

### Search Results

The computerized search yielded 433 references, of which 112 were included after abstract review. Citation search identified another 715 articles. Of the 827 articles that were obtained for full-text review, we excluded 808 articles based on the exclusion criteria. In particular, the result of Tuyns et al^48^ published in 1987 was replaced by Tuyns et al^32^ published in 1983, as it shared the same database. The result of De Stefani et al^49^ published in 2003 was replaced by De Stefani et al^40^ published in 2005, as the latter expanded the sample size based on the former population.

**FIGURE 1:**
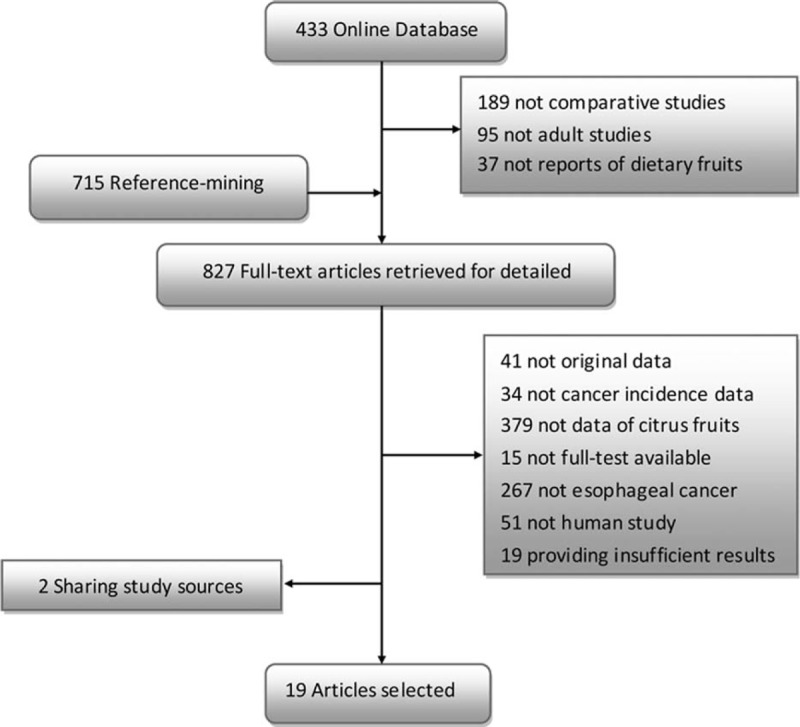
Flowchart of the searching and review of literatures.

A total of 19 articles were included in the meta-analysis, including 6 cohort studies^15,16,61,41-43^ and 13 case–control studies^9,10,17,18,32-39^ (Figure 1).

### Study Characteristics

Some details of the selected studies are shown in Tables 2 and 3. All articles were published in English. Six studies were conducted among residents of the United States,^18,31,33,35,36,39^ 1 in Italy,^38^ 2 in Japan,^16,43^ 2 in France,^32,37^ 3 in Europe,^9,41,42^ and the remaining 5 in China,^10^ Argentina,^34^ Switzerland,^17^ Uruguay,^32^ and the Netherlands.^15^ Two of the studies recruited participants in the 1980s, 5 in the 1990s, and 12 between 2000 and 2011.

**TABLE 2:**
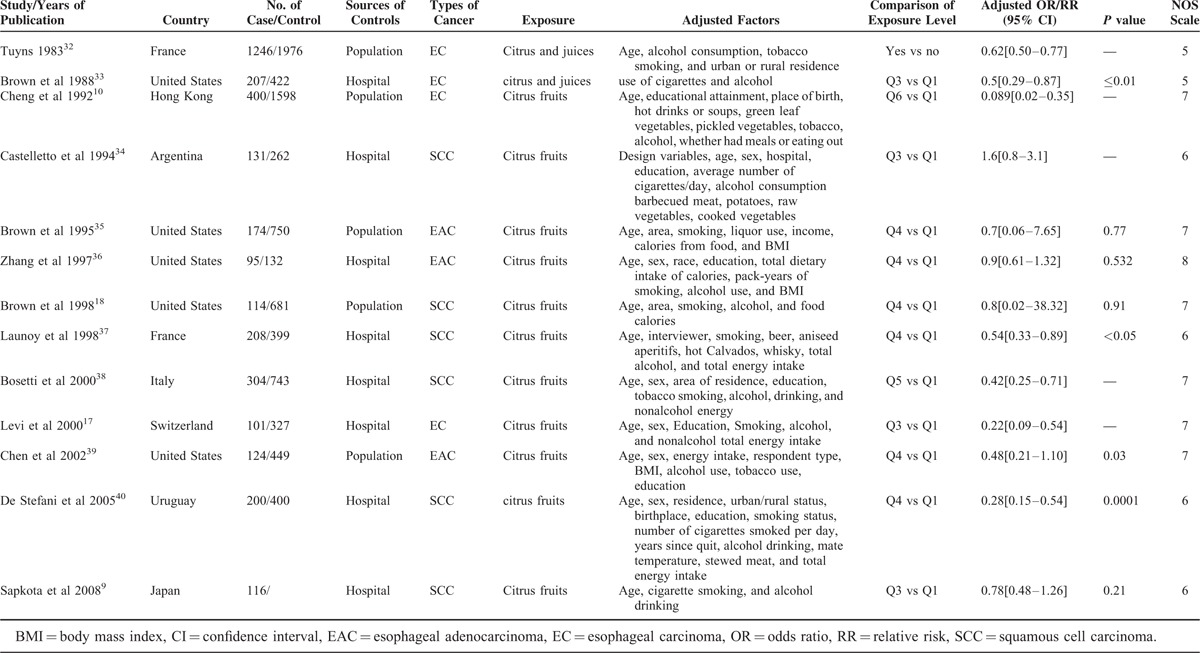
Summary of Case–Control Studies Included in the Meta-Analysis

**TABLE 3:**
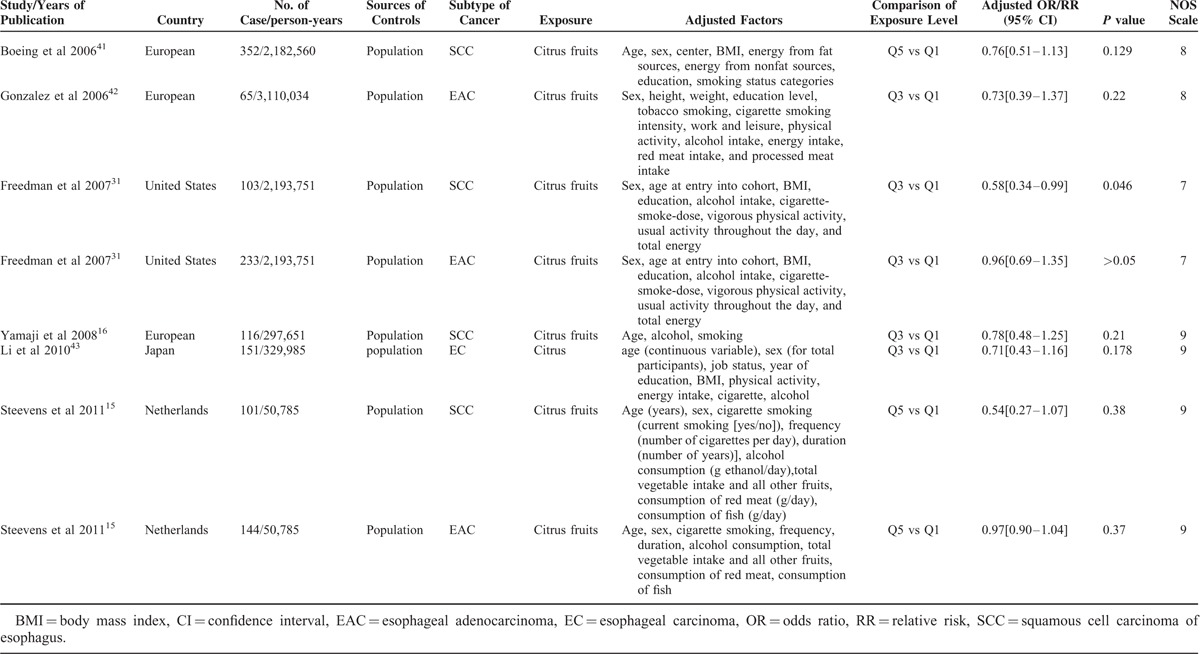
Summary of Cohort Studies Included in the Meta-Analysis

The factor of age was adjusted in all of the studies except Brown et al^50^ The confounding variables that were adjusted in different studies were presented in detail in Tables 2 and 3. For all of the studies, the relationship between intake of citrus fruits and esophageal cancer was not primary hypothesis and the citrus fruits were often included in a broader dietary evaluation. The ranges of adjusted ORs/RRs were from 0.089 to 1.6 and only 5 studies^31,32,33,37,39^ reached the usual threshold of *P* = 0.05 in the association between citrus fruits and esophageal cancer.

### Heterogeneity and Pooled Results

**FIGURE 2:**
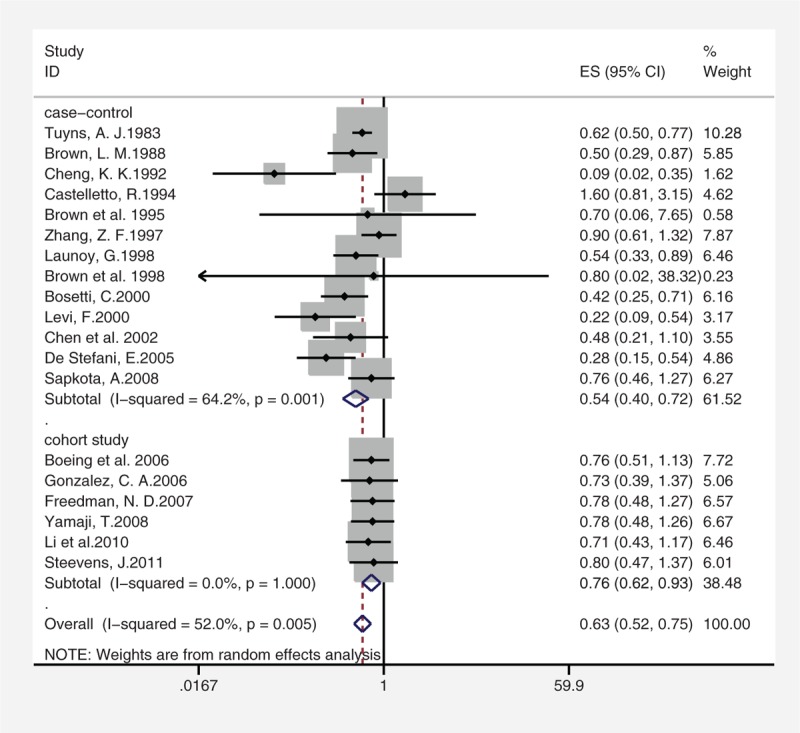
Summary estimates of the association between citrus intake and esophageal cancer risks sorted by effect estimate. CI = confidence interval; df = degree of freedom; chi2 = chi-square statistic; *I*^2^ = the percentage of total variation across studies that is due to heterogeneity rather than change; fixed = using fixed-effect model.

There was no significant heterogeneity among the study results (*I*^2^ = 52%; *P* = 0.005). Overall summary OR using the random-effects model showed a 37%, statistically significant reduction in risk of esophageal cancer associated with citrus fruits intake (summary OR = 0.63; 95% CI = 0.52–0.75). The subgroup of case–control studies (summary OR = 0.54; 95% CI = 0.4–0.72; *I*^2^ = 64.2%; *P* = 0.001) and the subgroup of cohort studies (summary OR = 0.76; 95% CI = 0.62–0.93; *I*^2^ = 0%; *P* = 1) showed a respective 46% and 24% statistically significant reduction in risk of esophageal cancer associated with citrus fruits intake (Figure 2). In subgroup analyses defined by study type, cancer subtype, geographical location, source of controls, research quality, and adjusted confounders, citrus intake was inversely associated with risk of esophageal cancer in most subgroups, with no evidence of significant heterogeneity between subgroups with meta-regression analyses. (Table 4).

**TABLE 4:**
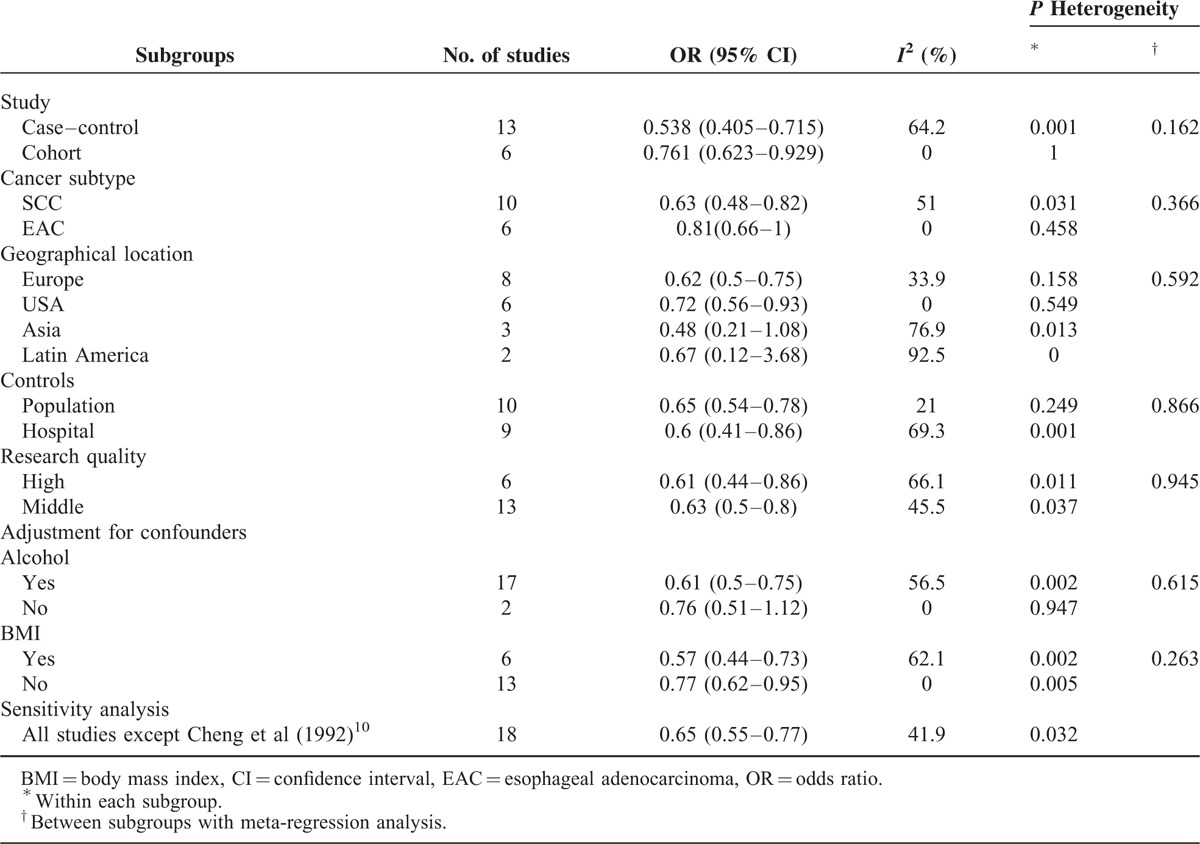
Subgroup Analyses of Citrus Intake and Risk of Esophageal Cancer, Sensitivity Analysis, Meta-Regression Analysis

### Publication Bias

**FIGURE 3:**
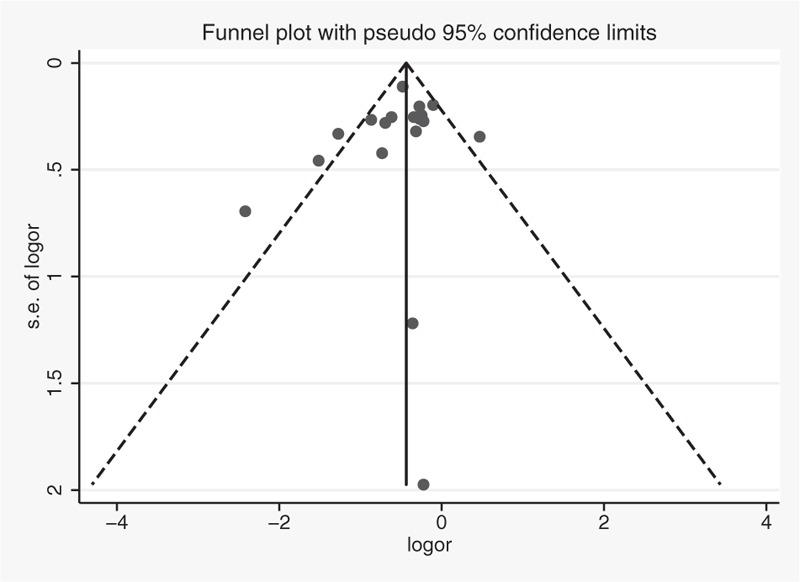
Funnel plot of studies evaluating the association between citrus fruit intake and esophageal cancer risks. Dot lines are 95% pseudo-confidence intervals. SE = standard error; OR = odds ratio.

No publication bias was observed in the selected studies. Visualization of Begg funnel plot was symmetrical (Figure 3). Formal testing using the Egger method supports the notion that there was no publication bias (intercept = −0.79, *P* = 0.288); however, the result of Begg test suggested an obvious publication bias (*P* = 0.046). And the outcome of trim and fill analysis demonstrated that there was no publication bias.

## DISCUSSION

The overall summary OR in our study presents an inverse association between citrus fruits and esophageal cancer (summary OR = 0.63; 95% CI = 0.52–0.75; *P* = 0). The result is supported by the strengths of our review, which includes a systematic literature search, strict selection criteria, comprehensive data abstraction, and rigorous statistical analysis. Additionally, the results of similar reviews about the association between citrus fruits and other cancers^26–29^ are encouraging.

Nevertheless, some limitations of our study should be taken into consideration. First, the considerable variables within observational studies made the outcome more likely to be suspicious.^50^ Furthermore, the included studies were evaluated to be low quality using the Grades of Recommendation, Assessment, Development and Evaluation system.^51^ These inherent drawbacks of observational study make outcomes more conservative.^52^ In addition, most of the included studies were not originally designed to evaluate citrus fruits and esophageal cancer, which possibly turns the pooled result into a simple summary.^50^ Measurement errors resulting from citrus intake should also be considered because of diversity of consumption patterns. Therefore, in the process of food intake measurement, various confounding factors made it difficult to obtain accurate dietary exposure information.^27^ Most case–control studies on diet are based on recent estimates of dietary intake,^15^ whereas the development of cancer after exposure to even a potent risk factor takes several decades.^53,54^

We found heterogeneity among the included studies, which decreases the quality of evidence to very low quality.^51^ Although rigorous criteria would make selective studies homogeneous, these could give rise to an inclusion bias.^55^ We excluded 2 studies^48,49^ sharing the same population, because the inclusion of duplicated data may lead to overestimation of exposure effects.^29,56^ Cancer deaths reflect failure of treatment as well as the occurrence of the cancer.^57^ Therefore, incidence rates are preferable as an early indicator of the impact of a risk factor. After careful screening of the eligible studies without CI or original data,^18,35^ we calculated the corresponding SE by the ORs and exact *P* values.^58^ The most appropriate way of handling the selection of studies is to perform sensitivity analyses with regard to the different possible entry criteria.^55^ Considering that the wide confidence internal of studies^18,35^ may obviously affect our outcome, we conducted sensitivity analysis.^29^ The analytic result showed that the studies have no apparent impact on the overall outcome. We also omitted 1 study^59^ that provided no citrus intake measurement. Methodology is a significant source of heterogeneity,^50^ so we performed subgroup analysis to verify the effect of study designs on heterogeneity. Figure 2 shows that study design causes heterogeneity. In the 6 cohort studies, the *I*^2^ of summary OR was 0%, because prospective studies can avoid recall and selection biases. In the 13 case–control studies, the *I*^2^ of summary OR was 64.2%. Both study designs demonstrate that citrus intake could reduce the incidence of esophageal cancer with summary OR 0.57 (CI 0.4–0.72), 0.76 (CI 0.62–0.93) for the case–control study and cohort study, respectively. The discrepancies between study results can be explained by recall and selection biases in case–control studies and by imprecise dietary measurements and limited variability of dietary intake in cohort studies.^27,60^ To further explore the source of heterogeneity, we conducted subgroups analyses and meta-regression analyses by many factors such as cancer subtypes, geographical location, source of controls, research quality, and adjusted confounders.

The pathogenesis and risk factors for different types of esophageal cancer^18,21^ vary widely, so exploring the impacts of citrus intake on these cancers is essential. Four of the included studies did not describe the specific cancer subtypes or included both subtypes. Table 4 shows the association between citrus and SCC (summary OR 0.63; CI 0.48–0.82) and EAC (summary OR 0.81; CI 0.66–1). The lack of overlapping confidence internal could partially explain the study heterogeneity. The forest plot (Figure 2) demonstrates that there is no overlap in CIs between 3 studies^10^ and the summary OR. Repeat meta-analysis of a new model excluded the study^10^ from all 19 selected articles was conducted.^61^ The level of heterogeneity decreased from high (*I*^2^ = 52%) to low (*I*^2^ = 0).

Citrus fruits include many bioactive components.^25,62^ Dietary antioxidants are emerging as potentially modifiable risk factors for EAC. High intake of beta-carotene may be associated with decreased risk of dysplastic Barrett esophagus, which is regarded as the precursor of EAC.^63^ Some studies^64,65^ showed that carotenoids may be responsible nutritional factors (as nutritional scavengers) in the development of different malignant diseases including esophageal cancer.^66,67^ Carotenoids may intervene in cancer-related molecular pathways and the expression proteins involved in cell proliferation, differentiation, apoptosis and angiogenesis, carcinogen detoxification, DNA damage, and repair.^68^ A related study indicates^69^ that a high intake of vitamin C is associated with a reduced risk of EAC and reflux esophagitis. Antioxidants may also play a role in the pathogenesis of reflux esophagitis and EAC and may be more important in terms of progression rather than initiation of the disease process^69^; however, low intake of vitamin C and E correlates significantly with the development of SCC as well as EAC in males.^70,71^ Regarding the mechanism, researchers think that vitamin C could enhance the EGCG- and TF3-induced apoptosis in SPC-A-1 and Eca-109 cells via MAPK pathways.^72^ Additionally, folate and other dietary methyl group factors are implicated in the etiology of EAC and its precursors. Folate is implicated in carcinogenesis via effects on DNA synthesis, repair, and methylation.^21,22^ Some studies indicate that flavanone intake is inversely associated with SCC risk and may account for the protective effect of fruit, especially citrus fruits, on esophageal cancer.^73,74^ Because citrus fruits account for 90% of flavanone intake, the findings of Rossi et al^73^ suggest that flavanones may play a role in the protective effect of citrus fruits on esophageal cancer. Therefore, the basic research of mechanisms flavanones protect against esophageal cancer are worth studying. Although the results are exciting, we have to taken in account the interaction between medicines and fruits. The research by Bailey et al^75^ suggested that there exist adverse reactions when grape is combined with some drugs.

Our review demonstrates that citrus fruit intake could reduce the incidence of esophageal cancer by 37% based on published results of epidemiologic studies. The trends are consistent between case–control studies and cohort studies; however, considering the drawbacks mentioned above, our conclusions should be taken cautiously. There are no relevant studies that provide explicit evidence for the inconsistency between SCC and EAC. The low quantity of EAC cases and the limitations of meta-analysis are responsible for the results. Therefore, larger studies with rigorous and prospective methodology should be considered to validate the association between citrus fruits and esophageal cancer. It is still unknown which components in citrus fruits have an effect on esophageal cancer prevention. Our conclusion may encourage researchers to further explore the protective elements and potential mechanisms, which may contribute to reducing the esophageal cancer risk. We hope further research will explore this issue.
